# Generation paths of online public opinion impact in public health emergency: a fuzzy-set qualitative comparative analysis based on Chinese data

**DOI:** 10.3389/fpubh.2024.1404587

**Published:** 2024-10-24

**Authors:** Teng Liu, Xiangming Hu, Qiangqiang Dong

**Affiliations:** ^1^Tourism College, Hainan Normal University, Haikou, Hainan, China; ^2^School of Public Administration, Beihang University, Beijing, China; ^3^School of Marxism, Zaozhuang University, Zaozhuang, Shandong, China

**Keywords:** online public opinion, public health emergencies, generation mechanism, QCA, public opinion risk

## Abstract

Public health emergencies can quickly provoke alarm and shock in the society, as well as generate high-impact online public opinion through network fermentation. Analyzing the generation mechanism of online public opinion in public health emergencies helps to explain its characteristics and laws. Based on information ecology theory, seven indicators from the four dimensions of information person, information, information technology, and information environment are extracted, and the analysis framework of public opinion impact of public health emergencies is constructed. Taking 40 cases from China as samples, fuzzy set qualitative comparative analysis (fsQCA) is used to investigate the generation path and mechanism of online public opinion impact in public health emergency. The results suggest that: information person and information technology are the core conditions for the generation of high-impact online public opinion, but the harm level contained in the information itself is not sensitive to the generation of public opinion impact; there are four generation paths and three types that drive the generation of high-impact online public opinion in public health emergencies. This work enriches the cognition of the causality of public opinion impact in public health emergencies from the perspective of configuration, and clearly shows which combination of variables leads to high-impact online public opinion, and helps to prevent and reduce the risk of public opinion.

## 1 Introduction

With the popularity of the internet and the expansion scale of internet users, the key role of online public opinion for the development of human society has been strengthened. According to the 50th China Statistical Report on Internet Development published by China Internet Network Information Center (CNNIC), up to June 2023, China had 1.079 billion internet users, and the internet penetration rate has reached 76.4%. The Internet brings scattered people together, which makes huge changes in the way of information dissemination. The trend of decentralization of online information dissemination is becoming obvious, while the dominant role of traditional media in public opinion is gradually declining. The new media based on the internet has become a new carrier of public opinion, represented by social media, live streaming and short video platforms. Compared to the traditional media, which has poor public opinion expression and low participation, the new media has become the public domain of public opinion expression because of its low communication threshold, fast speed and wide range (1). The public space, which can form public opinions, allows free communication and debate, has emerged (2).

In the internet age, online public opinion covers all aspects of our lives. While according to the content of public opinion, it can be divided into positive and negative public opinion (3). Several studies question the effectiveness of public opinion. As the main body of public opinion, people are easily guided and confused by elite groups and opinion leaders, together with the obscurity and complexity of public opinion itself, which all harm the objectivity and authenticity of public opinion (4). On the contrary, public opinion is not useless, some rational cognition and judgment of the public can be formed through limited information. The goal of environmental governance by online public opinion is to create positive public opinion and remove negative public opinion, so that online public opinion environment is presenting in a new form (5), and there are still many challenges and problems associated with building an online public opinion environment. If the online public opinion is not reasonably managed and evacuated, it may hurt society and the internet environment. Given the dual influence of online public opinion, this paper examined the dominant factors influencing online public opinion and how to prevent and reduce public opinion risk.

Moreover, contemporary society has entered the risk society. As risk disasters accompany the process of human society, the western theoretical community has started to study risky societies at an early stage, and has developed a relatively sound theoretical system (6). Unfortunately, the risk society is further deepened by public health emergencies, public health diseases have been a major threat to human health development, but along with the entire human history process. In recent years, major public health emergencies such as SARS, monkeypox, avian influenza, H1N1, Ebola, Zika, and other have occurred frequently, the outbreak of COVID-19 is particularly serious (7). Unlike other types of emergencies, public health emergencies are unpredictable, rapidly spreading and socially threatening, with no fixed population, easy-to-occur areas and ways of occurrence, which aggravate social risks, and the superposition of online public opinion risks and social risks triggered by network communication, which in turn derives new risks (8). The risk amplification effect of the internet has been dramatically accentuated, and it is not uncommon for individual cases and regional events to ferment into national or even global public opinion events. As it involves the life safety and physical health of every member of the public, information related to epidemics will quickly dominate the hot searches on the internet platform, triggering public attention and discussion. As a new social power and public opinion space, internet platform has become a platform and carrier for the public to express their views on specific events. Especially when public health emergencies occur, a large amount of true and false public opinion information emerges instantly and geometrically fissions, forming group polarization phenomenon, and even triggering secondary public opinion and offline group events, which poses a serious threat to social harmony and stability (9).

Online public opinion is a recurring theme in academic research (10), with the research exploring the impact of different factors on online public opinion. Our study attempts to answer the following research questions: (1) what factors are primarily responsible for the impact of online public opinion in public health emergency? (2) What is the generation paths of high-impact online public opinion? (3) What are the unique characteristics of the evolution of online public opinion influence? and (4) How to prevent and reduce the risk of public opinion? To address these issues, in the context of frequent public health emergencies as well as the downward shift of the focus of information dissemination in the era of new media, where the public and the mass media play a dominate role in the cognition and discussion of social issues, this study explores the influence factors of high-impact online public opinion. Qualitative comparative analysis (QCA) is employed in the study, the essence of this analysis method is to detect the configuration of causal conditions that lead to results of interest, and QCA offers an appropriate way to check which configurations of conditions best explain the generation of high-impact online public opinion, so that follow-up questions can be carried out. Specifically, we collected and adopted data from China internet social hot spot aggregation platform, using 40 typical public health emergencies as samples, we extract the four dimensions of information person, information, information technology and information environment, analyze seven driving factors, including internet users’ attention (IA), opinion leader dissemination (OD), government intervention (GI), hazard level (HL), network platform participation (NP), the government public opinion evacuation environment (EE), and the social opinion environment (SE), all of which shape the generation mechanism of online public opinion impact in public health emergency. According to the information ecology theory, the generation public opinion influence is systematic and complex. When the factors are combined with each other, we determine four different combinations to obtain the generation of high-impact public opinion. Our research results can better reflect the generation path of high-impact online public opinion in public health emergency than previous research results, and provide targeted suggestions for preventing and reducing the risk of public opinion.

Our study offers several contributions. First, we provide a targeted analysis of the special event of public health emergencies, which is a special field of emergencies. Public health emergencies and public emergencies, which are different in nature and characteristics, and thus the public opinions generated are also different. Second, we identify multiple influencing factors for the high-impact online public opinion of public health emergencies, expanding the scope of application of online public opinion research. Third, we demonstrate the configuration solutions between the interactions of the influencing factors of online public opinion, which is more suitable for preventing and reducing the risk of public opinion in the complex online public opinion environment, and thus maintaining a rational communication order.

## 2 Theoretical framework

### 2.1 Online public opinion

For Online public opinion is the sum of values and emotional tendencies expressed by the public through the Internet, which reflects the focus of public opinion and social situation. The pressure of public opinion is an important part that the government need to consider in order to avoid conflicts and obtain public support (11). Online public opinion dissemination system is a complex system based on causality. To demonstrate the generation mechanism of online public opinion dissemination, it needs to make effective subdivision of the influencing factors of online public opinion dissemination, to parse the complex system causality by analyzing the internal dynamic connection. Internet users, media, and government are the three main bodies of online public opinion dissemination system (12). Specifically, the participation of internet users and opinion leaders, the number of media audiences, the frequency of media reports, government attention, crisis warning mechanism, and other factors have an important impact on the online public opinion dissemination (13). In addition, response time, responsiveness, and government transparency also have an impact on public sentiment and public opinion dissemination in emergency situations (14).

Emergency online public opinion dissemination system is influenced by multiple and complex factors in social systems. Existing studies have analyzed the internal and external influencing factors of online public opinion, which provide us with profound understanding. However, there is a lack of research in public health opinion of public health emergency, and the interaction and combination of influence factors are relatively insufficient.

### 2.2 Information ecology theory

Online public opinion dissemination systems are often regarded as complex information ecosystems (15). Information ecology theory lays a good theoretical foundation for the study of multiple influencing factors of online public opinion dissemination from a holistic perspective. The concept of information ecology is the integration of ecology and informatics, it was originally used to investigate the flow of information in the organizations, where information did not operate independently but was affected by the system ecology. Information ecology is the science of studying the law of information, when studying the interaction of many different phenomena, it is necessary to analyze problems with a systematic view (16). A new information ecology theory has been further developed, which defines information ecology as a specific environmental system composed of people, practice, values, and technologies, and states that the focus of information ecosystem is not technology itself, but people (17). Moreover, there are strong interrelationships and dependencies among the different parts of information ecosystems, technologies, actors, environments and value orientations work together to constitute a complex system (18).

It is generally acknowledged by the academic community that information ecology theory is based on information ecosystems as research objects to analyze the interaction relationship of various elements within a system (19). To understand which configuration solutions have greater impact on online public opinion, a unified framework is needed, which incorporates multiple elements and specific environmental conditions. For this, the theoretical framework of information ecology is used to master the internal law of the development of information environment, deconstruct the dynamic changes of human social information environment, and make the information environment, especially the public opinion environment develop in a beneficial direction to human beings. Since information ecology theory is a broad concept, it integrates information person as subjects, while information, information technology and information environment as objects into a systematic visual threshold, each component of it needs to be excavated more deeply, which has its own established literature.

#### 2.2.1 Information person

Based on the perspective of information person factor in the information ecology theory, exploring highly complex human behaviors and analyzing user behavior laws are the core topics of research on emergency online public opinion dissemination (20). The information person factor is the subject of public opinion and the important component of the information ecosystem, which are people or organizations that express cognition, emotions, attitudes, opinions, statements and other remarks in cyberspace, mainly including internet users, network opinion leaders and government. Information person can interact with each other.

In the process of online public opinion dissemination in public health emergencies, internet users receive public opinion information and express their perceptions and attitudes toward the events in the internet, with the dual identities of information receivers and information disseminators. In the new media environment, internet users have become the largest and most active group in the network system. In the process of information exchange of online public opinion, internet users will resonate and converge as a group force due to the existence of cluster mentality, and directly drive the dissemination of online public opinion through comments, likes and retweets. Research shows that the active degree of internet users shows a positive correlation with the impact of online public opinion, and their attention to public opinion events is the main group force driving the spread of online public opinion and forming high-impact online public opinion (21). Therefore, the activity of internet users has an important driving role in the dissemination of online public opinion in public health emergencies.

Network opinion leaders, as active members who often provide information and exert influence on others in interpersonal communication networks, have a relatively stable and large information audience, occupy the accurate push resources of online platforms based on technical algorithms, and have a strong influence on the trend and speed of online public opinion dissemination. The speech and activeness of this group influence the generation of online public opinions and the dissemination of public opinion information, and play an aggravating and guiding role in the generation and dissemination of online public opinion in public health emergencies (22).

The government regulates and controls the development of online public opinion, as well as the information released by official media consisting of government units, central media and other authoritative institutions. Based on the trust of the government and the authority of the governance subject of social risk events, information with extensive credibility is released. Its strong dissemination and influence can squeeze out the space for deviant information, resolve public opinion crises in accordance with the law, reduce negative impacts, correct public opinion information, and orderly guide the promotion of a healthy public opinion ecosystem. It has been pointed out that timely and effective intervention and public voice of relevant departments can calm down negative social emotions and have a positive effect on public opinion mitigation (23).

#### 2.2.2 Information

As the ontology of public opinion, information is the sum of attitudes and emotions expressed by the participants of public opinion on hot topics, which is the root cause of public opinion. Lippmann pointed out that public opinion is developed from public interest, and whether the information and content of public opinion can arouse users’ interest is an important factor influencing their communication behavior. In addition, the degree of disclosure and credibility of information about an event also has an important influence on the development of public opinion (24).

Public health emergency has a real impact on public life safety and health, and the relevant information is very likely to mobilize public attention and generate online buzz. The outbreak of emotions, attitudes and intentions generated by high attention and online discussions constitutes a necessary condition for the generation of online public opinion events (25). With the advantages of high timeliness, interactivity and flatness, internet information has become the important way for the public to obtain information about public health emergency, while its instantaneous, individualized, and fragmented characteristics easily lead to distortion of the information carried, and in order to increase the heat and traffic, the importance and harm of public health events are expanded or one-sidedly disseminated, thus further causing amplification of risk communication and biased public perception (26). The information factors such as the hazard level contained in the information of public health emergency will become the focus of attention of online public opinion dissemination and the key elements of information text interpretation, coding and construction in the process of generation.

#### 2.2.3 Information technology

Information technology mainly refers to online media. Over the past decade, online media such as Twitter, Facebook, Microblog, TikTok, and WeChat have gradually become integrated into people’s lives and gradually blurred the boundaries of human society, and emergencies have accelerated this phenomenon. Not only internet users, but also more and more news media and government organizations are using social media for their work.

The participation of online media catalyzes the development of online public opinion. Online media reports on hot social events, realizing the dissemination of public opinion information in multiple directions, at multiple levels, and in many aspects, builds an information cultivation soil and amplification station for the dissemination of public health emergencies. Online public opinion information has become a sensitive tentacle for perceiving social and public opinion, and online media has become a platform for two-way communication and timely feedback in public health emergency (27).

In public health emergency, due to the lag of government response to disposal and information disclosure, the public generates various doubts and speculations, which will express tendentious emotions, opinions and remarks on online media platforms for concerns and exert certain influences. Driven by the interest chain of heat, traffic, fan increase and cash, online media platforms pursue the “signal value” brought by the high-impact public opinion and the event itself, and use platform resources to fatten up and add material to further promote the influence of public opinion on the event. Online media platforms are not only the petri dish for the influence of online public opinion in public health emergency, but also the weathervane for the influence of public opinion based on the feedback of public opinion information, and the number of network platforms reporting on the event can be used as a barometer for the influence of public opinion (28).

#### 2.2.4 Information environment

Information environment, it mainly refers to the public opinion environment, which is the external environment and space for the dissemination of online public opinion. In public health emergency, it mainly includes the public opinion evacuation environment constructed by information environment control subjects and the social opinion environment naturally formed by public opinion. Government authorities, online media platforms, health and medical research institutions and public health professionals constitute the control subjects of information environment, among which government authorities occupy the core position of control subjects (29).

External intervention in the dissemination and development of public opinion is mainly carried out by the government through organizing official central media to release information, online media platforms to restrict negative and false statements, health and medical research institutions, together with public health professionals to popularize public health and safety knowledge (30, 31), which are the exogenous driving force for the evolution, development and resolution of public opinion. The specific countermeasures and strength of the government’s public opinion evacuation have significant influence on the formation of the public opinion evacuation environment. The public opinion environment can reflect the development trend of public opinion, the attention degree of information subjects to public opinion information, the duration of public opinion and the influence of public opinion events, and it is the endogenous driving force for the evolution and development of public opinion. During the duration of public opinion, the amount of information related to public opinion can reflect the influence of public opinion environment on the information subject.

### 2.3 Analytical framework

Accordingly, on the basis of the previous theoretical discussion, this paper firstly identifies the preconditions for the generation of online public opinion from the perspective of system and based on the theoretical framework of information ecosystem, with information person (public opinion subject), information (public opinion ontology), information technology (public opinion platform), and information environment (public opinion environment) as the four analytical dimensions, and extracts seven preconditions affecting online public opinion in public health emergencies: internet users’ attention (IA), opinion leader dissemination (OD), and government intervention (GI) correspond to the degree of information person’s attention, the hazard level (HL) of public health emergencies corresponds to the content elements embedded in the information, network platform participation (NP) corresponds to information technology, and the government public opinion evacuation environment (EE) and the social opinion environment (SE) correspond to the information environment. Meanwhile, the outcome variable is online public opinion impact generation.

**Figure 1:**
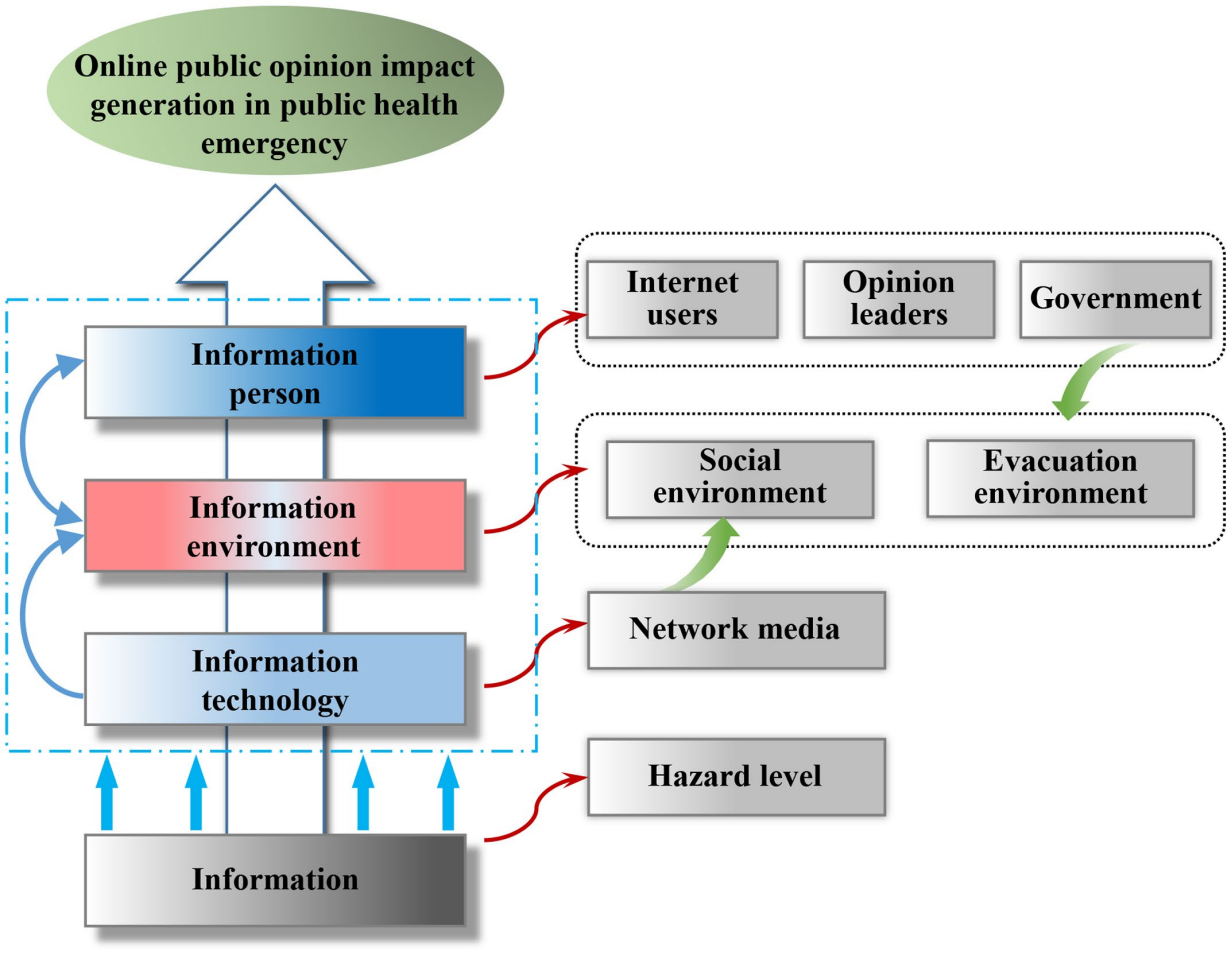
Analytical framework of the configuration online public opinion impact in public health emergency.

This paper focuses on the cognition of the causality of the generation of high-impact public opinion and not high-impact public opinion, emphasizes the interaction and combination of factors in the system, and studies how the factors can jointly drive the generation of high-impact online public opinion of public health emergencies through configuration solutions. In summary, the analytical framework of this paper is constructed, as shown in Figure 1.

## 3 Materials and methods

### 3.1 Sample

Due to the large number of users of Twitter, Facebook, Microblog, TikTok, WeChat, and other online media, these online media are often used by the public to spread public opinion on emergencies. Current research is increasingly focused on obtaining information on public opinion, attitudes, and key factors related to various research subjects through various online media. Several studies have shown that to analysis of tweets and images about Zika virus in Twitter and Instagram to study the dissemination of public opinion information, it can be found that news media, public health agencies and internet users are the most obvious and frequent sources and disseminators (32). Some studies have also examined the public opinion information dissemination behavior through Microblog message posting, commenting and retweeting data (33).

Microblog and WeChat are the widely used online media platforms in China (34–36), its data has become a popular source of academic research, providing researchers with rich materials and in-depth insights. According to the financial report released by Tencent Holdings and Sina Microblog, by the end of December 2023, the combined monthly active accounts of WeChat and WeChat international numbered 1.343 billion, while Microblog had 598 million monthly active users. Therefore, researchers can obtain high-quality raw data, and aggregated user opinions can reflect the online public opinion of an event. Due to the wide impact of public health emergencies, individuals do not have the ability to collect all the data from the Internet and data analysis platforms, so the Zhiwei data analysis platform is used as an event impact index analysis tool (37). Zhiwei Data are one of the most commonly used Internet hotspot aggregation platforms in China. Compared with data analysis platforms such as China Percent Technology and Qingbo Big Data, Zhiwei Data is a specialized analysis tool for popular events on the Internet, especially good at building a complete ecology of discovery, tracking, mining and prediction of social hotspot events based on massive Internet data, which is applicable to individuals, enterprises and governments. The main data sources of Zhiwei Data Analytics Platform are microblogs, WeChat and online media. The event influence index is calculated by adding up the event impacts in the self-media (mainly Weibo and WeChat) and online media, and then normalizing the event impacts to get an event impact index ranging from 0 to 100. Individuals can filter public health emergencies according to the keywords of the event, and can query the main media that released the information, the trend of the proportion of the public opinion field, the speed of dissemination of the event, the duration of the event, and other public opinion field data, which can form an effective evaluation criterion of the influence of the event and conduct comparative analysis of similar events. The 79th executive meeting of the State Council of China adopted the Overall Emergency Plan for National Public Emergencies, which was promulgated and implemented on January 8, 2006. It is clearly pointed out that public health incidents mainly include the epidemic situation of infectious diseases epidemic, mass diseases of unknown causes, food safety and occupational poisonings, animal epidemics, and other incidents that seriously affect public health and life safety. In this study, the high-impact public health emergencies in the database of Zhiwei data platform from 2016 to 2023 as the basic case base, and the cases were cross-validated and supplemented by CNKI, Baidu index, and Microblog, Ant Square software, etc. In order to reflect the principle of influence, typicality and diversity of cases, four cases of the same subspecies of avian influenza outbreak were deleted, one case of norovirus related cases with similar time but different regions was deleted, and three cases of food poisoning in the basic case database were added (food poisoning of 100 employees of a furniture factory in Shanghai, vomiting and diarrhea in a community in Shenyang, and Suspected food poisoning occurred in a snack bar in Guangdong), as well as one environmental pollution incident (40,000 mu of fish and crabs died in Hongze Lake), one case of occupational and chemical poisoning incident (brucella infections at two veterinary research institutes under the CAAS). Finally, 40 typical events were finally selected as the research objects, including six cases of infectious disease epidemic, two cases of unknown-cause disease, nine cases of animal epidemics, 10 cases of food safety, four cases of occupational and chemical poisoning, and nine cases of drag safety and pollution, as shown in Table 1.

**Table 1:** Typical cases of public health emergencies.

No.	Year	Case	Type	Influence Index(II)	No.	Year	Case	Type	Influence Index (II)
1	2019	COVID-19 in Wuhan and other places	UD	100	21	2016	Formaldehyde poisoning of all junior students in a school in Jilin	OC	68.5
2	2018	Changsheng Biological Vaccine Fraud Incident	DP	94.1	22	2018	Multiple children vaccinated with expired vaccines in Shanxi	DP	68.3
3	2016	Unfrozen vaccines flow into 18 provinces	DP	86	23	2020	Bayan Nur confirmed a case of glandular plague	AE	68.1
4	2023	The rat head and duck neck incident in Jiangxi Industry Polytechnic College	FS	85.9	24	2021	Seven deaths due to harmful gas leakage from a food factory in Sichuan	OC	66.9
5	2020	Fake milk powder caused the reappearance of large-headed children	FS	82.2	25	2020	H5N1 avian influenza outbreak in Hunan	AE	66.6
6	2018	Pig, cattle and sheep epidemic outbreak in many places	AE	81.2	26	2019	Norovirus infection in Chaoyang District, Beijing	ID	65.6
7	2022	Pickled Chinese sauerkraut in dirt pits incidents	FS	80.2	27	2019	A patient diagnosed with glandular plague in Xilingole Meng	AE	65.5
8	2019	A school in Chengdu gave students moldy food caused discomfort	FS	78.6	28	2021	Anthrax deaths occurred in Shandong Province	ID	63.8
9	2016	Students’ abnormal health caused by the relocation of a middle school in Jiangsu	OC	76.2	29	2019	Sichuan children suspected of receiving expired vaccines	DP	63.2
10	2020	Heilongjiang “sour soup” poisoning incident	FS	75.1	30	2018	Food poisoning in a hotel in Guilin	FS	62.3
11	2019	11 enterprises suspected of detecting African swine fever	AE	73.9	31	2022	Henan confirmed 1 person infected with H3N8 avian influenza	AE	61.8
12	2019	A hospital in Hainan has been reported to sell fake HPV vaccines	DP	73.7	32	2018	Nearly 40,000 mu of fish and crabs died in Hongze Lake	DP	61.7
13	2019	145 children in Jiangsu province received oral expired vaccine	DP	72.9	33	2019	Brucella infections at two veterinary research institutes under the CAAS	OC	61.4
14	2021	Clenbuterol mutton appeared in Hebei province	FS	72.1	34	2019	Beijing confirmed 1 person infected with H5N6 avian influenza	AE	61.2
15	2023	Rice with essence event	FS	71.6	35	2020	H5N6 avian influenza outbreak in Xichong, Sichuan	AE	57.4
16	2019	The vaccine of a community health center was replaced by low-cost vaccine	DP	69.9	36	2019	Tianjin University of Technology students infected with norovirus	ID	57.3
17	2022	BYD factory is accused of pollution causing nosebleed of many children	DP	69.5	37	2018	African swine fever has been confirmed in Jizhou, Tianjin,	AE	57
18	2019	Tuberculosis epidemic in Taojiang, Hunan	ID	69.4	38	2020	Suspected food poisoning occurred in a snack bar in Guangdong	FS	48.6
19	2017	H3 influenza outbreak in Hong Kong	ID	69.3	39	2019	300 people vomiting and diarrhea in a community in Shenyang	UD	39.2
20	2016	The second case of Zhaika virus infection confirmed in China	ID	68.5	40	2016	Food poisoning of 100 employees of a furniture factory in Shanghai	FS	28.1

ID, Infectious diseases epidemic; UD, Unknown-cause disease; AE, Animal epidemic; FS, Food safety; OC, Occupational and chemical poisoning; DP, Drug safety and pollution.

### 3.2 Methods

Qualitative comparative analysis (QCA) is a configuration method based on set theory and fuzzy theory (38), which is particularly suitable for studying complex causality and multiple interactions (39). The generation of online public opinion impact in public health emergencies is influenced by the coupling effect of multiple complex factors, which is suitable for the QCA method. First, QCA can identify whether a single condition is necessary to achieve an outcome. Second, it can explore multiple sufficiency configurations associated with the same outcome, a situation called equilibrium, where a system can reach the same final state through different initial conditions and a variety of different paths. Third, QCA can check for causal asymmetry for high and not-high performance.

Qualitative comparative analysis is based on Boolean logic, mainly studying necessary conditions and sufficient conditions. In detail, necessary conditions are those that are present in all cases of outcome, and sufficient conditions are those that always produce a certain outcome when present (40). QCA results are interpreted according to consistency and coverage. Consistency refers to the extent to which similar causal configurations lead to outcomes, while coverage refers to the number of cases valid for a given combination. We show our results by the conventional notations: core conditions are present ● or absent ⊗, peripheral conditions are present ● or absent ⊗, and a blank space indicates that the condition is irrelevant whether it is presence or absence (41). Core conditions are those present in both parsimonious solutions and corresponding intermediate solutions. Peripheral conditions are those present intermediate solutions, but not in parsimonious solutions (42). Fuzzy-set QCA (fsQCA), unlike the crispy-set QCA (csQCA), and the multi-valued QCA (mvQCA), uses membership degree assignment, which improves the research quality. CSQCA can only handle two-point variables. Although mvQCA expands the scope of dichotomy to a certain extent, it is still based on deterministic multivalued sets rather than continuous fuzzy sets. FSQCA is more case-oriented and able to explain the causal factors in more detail. Therefore, we apply the fsQCA in study.

### 3.3 Measures for set membership

Too many variables will lead to individualization of cases, and the number of cases identified needs to form a well-balanced relationship with the condition variables. QCA technology could be suitable not limited to handle data from small and medium samples, and it has also been widely applied in research designs with large sample sizes (41). The sample size of our study belongs to the medium-sized sample, which is suitable for analysis by QCA method. A medium-sized sample means that the external validity of the case can be guaranteed, and the depth of the case and its uniqueness can also be retained. For a medium-sized sample, the usual selection of condition variables is 4–6 or 4–7 (43). Therefore, we determine that the seven condition variables met the requirements. The settings and measurement descriptions of the condition variables and outcome variables are shown in Table 2.

**Table 2:** Measurement descriptions for sets.

Type	Sets		Measurement descriptions
Condition variables	Information person	Internet users’ attention (IA)	Average speed of information dissemination during events
		Opinion leader dissemination (OD)	The total number of fans of the top five Microblog bloggers involved in the event discussion
		Government intervention (GI)	The proportion of participating central media in all media
	Information	Hazard level (HL)	Classified according to the “Emergency Plan for Public Health Emergencies” and the Law of “China on Prevention and Control of Infectious Diseases”
	Information technology	Network platform participation (NP)	The number of online media platforms for reporting
	Information environment	Evacuation environment (EE)	Refers to the government’s efforts to alleviate public opinion, represent sum of products of government feedback department level、feedback channels and times $Q=\sum L_{i}C_{j}N_{k}$ (*i* = 1,2…5; *j* = 1,2…5; *k* = 1,2…*n*)
		Social opinion environment (SE)	Number of Baidu related web pages during the duration of public opinion (Baidu Index)
Outcome variable	Online public opinion impact generation (II)		The total cumulative dissemination effect of the case in the media, which can be reflected by the influence index

Q refers to the government’s efforts to alleviate public opinion; L_i_ means government feedback department level, L_1_ (the central leadership or State Council) = 4, L_2_ (the national level) = 3, L_3_ (the provincial level) = 2, L_4_ (the city level) = 1, L_5_ (the county level) = 0.5; C_j_ means feedback channel, C_1_ (special meetings) = 4, C_2_ (press conferences) = 3, C_3_ (official media) = 2, C_4_ (competent leaders or department core experts) = 1, and C_5_ (relevant experts’ report) = 0.5; N_K_ means times.

### 3.4 Calibrations for set membership

Calibration is the process of transforming into set membership, the variables must be calibrated to generate values from 0 to 1 before using fsQCA for analysis. There are three methods to determine which qualitative anchors to use for scale measures, the first calibration method is to obtain substantial knowledge from pre-validated scale anchor points as the threshold values, the second relies on sample maximum, mean or midpoint, and minimum values when partial knowledge is available, and the third is to use the percentile of the sample without explicit theory and external knowledge as a guide (44).

Since there is a recognized basis and standard for classifying the hazard level of public health emergencies, the first calibration method can be used. Specifically, we use the four-value fuzzy set calibration method, which uses four calibration values of 1, 0.67, 0.33, and 0 to refer to “full in,” “more in than out,” “more out than in,” and “full out,” respectively. In the event hazard level, extremely significant (level I) is assigned a value of 1, significant (level II) is assigned a value of 0.67, large (level III) is assigned a value of 0.33, and general (level IV) is assigned a value of 0. For other variables, there is no explicit theory or external knowledge available, so the third method mentioned above can be used. Based on the data of the cases themselves, qualitative anchors representing three important threshold ranges of full in, crossover point, and full out are selected for calibration. The three qualitative anchors are taken from the upper and lower quartiles of the case data, which are the upper quartile, the median, and the lower quartile. Specifically, the three anchors of the variables, except for the calibrated hazard level, are set as fully in (75%), crossover point (50%), and fully out (25%). In addition, when a case has a membership score of exactly 0.5, it is usually recalibrated each set by adding a small constant (0.001) (41). When cases arise where the affiliation score is exactly 0.5 and cannot be categorized, the categorization is usually adjusted by adding 0.001 to the affiliation score. The calibration anchors for each fuzzy set, and descriptive statistics are shown in Table 3.

**Table 3:** Sets, calibrations and descriptive statistics.

Sets	Fuzzy set calibrations			Descriptive statistics			
	Fully in	Crossover	Fully out	Mean	SD	Min	Max
IA	22.50	7	2.25	30.70	67.01	1	298
OD	5,318	2,896	1136.75	6174.50	10630.39	0	52,987
GI	54.33	39.40	5.50	35.74	27.69	0	93.1
HL	1 0.67 0.33 0			0.26	0.30	0	1
NP	81	44	18.25	54.43	51.58	1	240
EE	17.75	12	5	37.59	149.19	1	965
SE	117,352	29,735	10123.50	141,105	316240.80	1,142	1,866,780
II	74.80	68.50	61.93	68.57	12.87	28.10	100

## 4 Results

### 4.1 Necessity conditions analysis

When a factor is always present when an outcome occurs, this factor is called a necessary condition for this outcome, from which the core conditions leading to the outcome can be initially determined. The impact factors of online public opinion generation in public health emergencies are mainly to analyze the necessity of condition variables and to examine consistency and coverage, consistency greater than 0.9 can be regarded as a necessary condition for an event to occur. Coverage is mainly used to determine the degree to which a conditional variable can interpret the outcome variable, and a larger value means a stronger explanation of the conditional variables. Table 4 presents the results of this analysis.

**Table 4:** Analysis of necessary conditions.

Sets	Outcome = public opinion impact generation		Outcome = ~public opinion impact generation	
	Consistency	Coverage	Consistency	Coverage
IA	0.845	0.914	0.210	0.223
~IA	0.282	0.267	0.919	0.853
OD	0.971	0.961	0.261	0.253
~OD	0.245	0.253	0.960	0.970
GI	0.822	0.816	0.319	0.310
~GI	0.305	0.314	0.811	0.817
HL	0.340	0.667	0.317	0.609
~HL	0.800	0.544	0.827	0.551
NP	0.895	0.924	0.247	0.250
~NP	0.274	0.271	0.925	0.896
EE	0.782	0.773	0.401	0.388
~EE	0.382	0.394	0.766	0.775
SE	0.713	0.730	0.374	0.375
~SE	0.389	0.388	0.730	0.714

~ means the absence of. For example: ~… = absence of high.

We can see from the Table 4 that when the outcome variable is set to “public opinion impact generation,” the consistency of OD reaches 0.971, indicating higher opinion leader dissemination is a necessary condition for the generation of public opinion influence in public health emergencies. At the same time, the coverage of OD reached 0.961, indicating that 96.1% of public health emergencies had the intervention and impact of dissemination of opinion leaders. In addition, the consistency of IA, GI and NP is 0.845, 0.821, and 0.895, respectively, which exceeds 0.8. All of them can be regarded as sufficient conditions for the generation of high-impact online public opinion in public health emergencies. From the necessity test of a single variable, we can find that IA, OD, GI, and NP can independently trigger the generation of high-impact online public opinion, which shows that information person and information technology have a significant impact on the generation of high influence of online public opinion.

When the outcome variable is set to “~public opinion impact generation,” the consistency of ~IA, ~ OD and ~ NP is higher than 0.9, which are all necessary conditions for the generation of not high-impact online public opinion in public health emergencies and have stronger explanatory power. Besides, the consistency of ~GI and ~ HL is greater than 0.8, which is a sufficient condition for the generation of not high-impact online public opinion. According to the information ecology theory, the generation of online public opinion impact is systematic and complex, and the synergistic influence of various factors such as information person, information, information technology and information environment needs to be further analyzed.

### 4.2 Sufficiency analysis for online public opinion

We use fsQCA3.0 software to deal with the standardized data, construct the truth table and conduct a sufficiency analysis. QCA analysis usually provides three solutions: complex solution, parsimonious solution and intermediate solution. The intermediate solution is often considered to be the preferred solution because its complexity is moderate and reasonable, and the necessity conditions of the intermediate solution cannot be eliminated, so it is often considered as the preferred solution. Referring to the existing research, this paper selects the intermediate solution for analysis (45). Referring to existing studies, we use a frequency benchmark ≥ 1, raw consistency benchmark ≥ 0.8, and a proportional reduction in inconsistency (PRI) ≥ 0.70 (46). Using these standards, we obtain the truth table and identified four configuration solutions that can lead to high-impact online public opinion. The overall solution coverage is 0.725, indicating that these configurations can explain about 72.5% of the high-impact generation outcomes of online public opinion, and overall solution consistency is 0.994, which can cover and explains the high-impact generation of online public opinion. We further identified six configuration solutions that can lead to low-impact generation of online public opinion. The overall solution consistency is 0.977, with a coverage of 0.784.

We analyze four configuration solutions of high-impact online public opinion horizontally and found that they all have the same core conditions. Based on the difference of the four configuration solutions, three types of online public opinion generation with high impact in public health emergencies can be divided: information environment driven in a crisis-free situation, dual EE-SE driven in a non-sensitive crisis situation, and a single-driven type of crisis situation.

#### 4.2.1 Configurations for high-impact online public opinion

In Table 5, we display results for four configuration solutions where the solutions are sufficient for high-impact online public opinion with high solution consistency and solution coverage (0.994 and 0.725, respectively) and that satisfied consistency and coverage of each solution.

**Table 5:** Configuration for public opinion generation (fsQCA).

Sets	Configuration for high-impact public opinion generation				Configuration for not high-impact public opinion generation						
	S1a	S1b	S2	S3	S4	S5	S6	S7	S8	S9	S10
IA	●	●	●	●	⊗	⊗	⊗	⊗	⊗	⊗	●
OD	●	●	●	●	⊗	⊗	⊗	⊗	⊗	⊗	●
GI			●	⊗	⊗	⊗		⊗	⊗	●	⊗
HL	⊗	⊗		●	⊗	⊗	⊗			⊗	●
NP	●	●	●	⊗	⊗	⊗	⊗	⊗	⊗	●	⊗
EE	●	⊗	●	⊗	⊗		⊗	⊗	●		●
SE	⊗	●	●	⊗		⊗	●	●	⊗	⊗	⊗
Consistency	0.983	0.992	0.997	0.982	0.987	1	0.953	0.971	1	0.982	0.998
Raw coverage	0.265	0.196	0.450	0.080	0.542	0.497	0.297	0.273	0.241	0.139	0.056
Unique coverage	0.128	0.087	0.279	0.040	0.009	0.015	0.031	0.022	0.017	0.045	0.011
Overall solution consistency	0.994				0.977						
Overall solution coverage	0.725				0.784						

Solution 1 (S1) is information environment driven in a crisis-free situation. This type is the main type and consists of combination S1a and S1b in which the internet users’ attention, opinion leader dissemination, and network platform participation are core conditions, and the lack of hazard level plays a supplementary role, government intervention is irrelevant. Only the information environment is different. It shows that even if the public health emergencies with low hazard level can attract the high attention of informants represented by internet users and online opinion leaders, and more information technology platforms participate in the reporting, although they lack the attention of the central media, they can form a high-impact online public opinion in both a good evacuation environment and an active social opinion environment. The consistency of S1a and S1b reached 0.983 and 0.992 respectively, and the coverage rates were 0.265 and 0.196, respectively. This type of events mainly focused on public health emergencies closely related to public life, such as the rat head and duck neck incident in Jiangxi Industry Polytechnic College, a school in Chengdu gave students moldy food caused discomfort, 11 enterprises suspected of detecting African swine fever, and Clenbuterol mutton appeared in Hebei province.

Solution 2 (S2) is dual EE-SE driven in a non-sensitive crisis situation. In addition to the core roles played by internet users’ attention, opinion leader’s dissemination, and network platform participation, evacuation environment and social opinion environment are marginal conditions that play supplementary roles, and event hazard level is an irrelevant condition. Specifically, regardless of the hazard level of public health emergencies, as long as the high attention of information person and the participation of more network platforms are aroused, most public health emergencies can attract widespread attention in the society, and the high influence of online public opinion can be formed through the drive of public opinion evacuation environment constructed by governments at all levels, public health departments and experts, as well as the social public opinion environment formed naturally by public opinion. The consistency of S2 reaches 0.997, and the coverage rates is 0.450. Dual EE-SE driven in a non-sensitive crisis situations are mainly focused on medical safety and epidemic infectious diseases, because public health emergencies have an important impact on life safety and physical health, which is easy to trigger public panic and form a public opinion environment. Typical examples include the COVID-19 outbreak in Wuhan and other places in 2019, Changsheng Biological Vaccine Fraud Incident in 2018, and Unfrozen vaccines flow into 18 provinces in 2016.

Solution 3 (S3) is a single-driven type of crisis situation. In S3, the internet users’ attention and opinion leader dissemination play core roles, and the lack of government intervention and the lack of participation of network platforms also play a core role. The hazard level of the event plays a supplementary role, and the lack of evacuation environment and the lack of social opinion environment also play a supplementary role. For public health emergencies with high hazard level, based on the joint effect of the two core conditions of high online public opinion influence, high internet users’ attention and high opinion leader dissemination, even if the participation of central media and network platform is low, the evacuation environment for public opinion is lack and the social opinion environment is not active, it can still form high-impact online public opinion. The single-driven type of crisis situation mainly focuses on small-scale public health emergencies of infectious diseases with high hazard level, such as Beijing confirmed one person infected with H5N6 avian influenza in 2019, Bayan Nur confirmed a case of glandular plague in 2020, Brucella infections at two veterinary research institutes under the CAAS in 2019.

From Table 5, we can find that in public health emergencies, the core pathways of high-impact generation of online public opinion are S1 and S2, with the raw coverage rate higher than 45%. By integrating the two core pathways, we can get the formula of the generation type of high-impact of online public opinion of public health emergencies: public opinion influence = A*B*E (C + ~D) (F + G). From the simplified formula, it can be seen that in public health emergencies, information person and information technology are still proved to be the necessary conditions for the high-impact pathway, and both the information environment of public opinion evacuation environment and social opinion environment also play the important role of supplement conditions in the combination of conditions, while the impact of the hazard level factors contained in the information is contrary to traditional cognition.

#### 4.2.2 Configurations for not high-impact online public opinion

Besides investigating the configurations for high-impact online public opinion, we also display results for six configurational solutions for not high-impact online public opinion in Table 5, with high solution consistency and solution coverage (0.977 and 0.784, respectively) and that satisfied consistency and coverage of each solution. At the same time, Solution 4 and 5 are the core pathways for the generation of not high-impact public opinion in public health emergencies. They both have a raw coverage rate higher than 45%. By integrating the core pathways, the formula for generating not high public opinion can be simplified as follows: ~A* ~ B* ~ C* ~ D* ~ E*(~F + ~G). It can be seen that in public health emergencies, information person and information technology are still proved to be the necessary conditions for the not high-impact pathway, public opinion evacuation environment and social opinion environment included in the information environment, as well as hazard level also play the role of necessary conditions in the combination of conditions.

### 4.3 Robustness checks

In order to avoid the randomness and sensitivity of the results, robustness test is necessary in QCA analysis. The specific robustness test of set theory mainly includes adjusting the calibration threshold, modifying the frequency of cases, resetting the consistency threshold, adding other conditions, supplementing or eliminating cases, etc. Based on the existing research experience, we carried out the robustness test by eliminating some cases. According to the indicator of generating public opinion impact in the outcome variable, cases were excluded. Three qualitative anchor points (74.80, 68.50, and 61.93) were used to divide 40 cases into four groups. Cases 1–10 are grouped together, and cases 5, 6, and 7 were excluded; cases 11–21 are a group, excluding cases 15–17; cases 22–30 are grouped together, excluding cases 25, 26, and 27; cases 31–40 are grouped together, excluding cases 35, 36, and 37. A total of 12 cases are eliminated, and then we analyzed the remaining case sets again. After the robustness test and analysis, the conclusion is basically consistent with the original conclusion, and can confirm each other, indicating that the research results remained robust.

## 5 Discussion

### 5.1 Research conclusions

Through qualitative comparative analysis of fuzzy sets of research cases, we extract and identify the core conditions and configurational solutions for the generation of high-impact online public opinion in public health emergencies, we found that the generation and evolution of the impact of online public opinion basically follow the action mechanism and common evolutionary characteristics of information ecology theory and online public opinion dissemination theory, but also have their unique evolutionary characteristics.

Information person and information technology are the key conditions for the generation of high-impact online public opinion in public health emergencies. From the necessity measure of a single variable, we can observe that higher internet users’ attention, opinion leaders’ communication power and online platforms’ participation are necessary conditions for the generation of high-impact online public opinion in public health emergencies. Meanwhile, from the configurational solutions we can also find that the three are the core conditions for the generation of high-impact public opinion, which can prove that the information person and information technology are the key forces for the generation of online public opinion impact of public health emergencies. With the development of information technology, online media characterized by mobile, extensive, civilian, social, and interactive has emerged, and the survey found that online media gradually dominated the competition with central media, which can indicate that opinion leaders and network media platforms also have strong enough driving force in online public opinion impact generation. Comparing the two core combination pathways, both the information environment of public opinion evacuation environment and social opinion environment can generate high public opinion impact. If the government involvement is higher and the evacuation degree is greater, then the information environment that plays a key role in high-impact online public opinion will be constructed by the government. Although government public opinion evacuation environment is not a necessary condition for public opinion impact generation, public health emergencies with longer duration of public opinion and greater difficulty of government public opinion evacuation are often found in the pathway with higher degree of government intervention.

However, from the perspective of public opinion risk preventing and reducing, configuration S2 is the best, S3 is the worst, S1a is better than S1b. Traditionally, the higher hazard level of event, the greater the casualties and economic losses, the longer the time of government public opinion risk relief, the more the online media will continue to question the progress of events, and the number of network platform participation and the duration of reporting will greatly increase, thus promoting the generation of high-impact online public opinion. However, the study found that the generation of high-impact online public opinion is not sensitive to the hazard level of the event itself, indicating that the information elements of high-impact public opinion on the network causing public health emergencies are multivariate. Therefore, the contrary should not simply be based on the hazard level of public health emergencies as a predictive measure of public opinion risk.

### 5.2 Theoretical implications

First, based on the information ecology theory and the online public opinion dissemination theory, we constructed an analytical framework of online public opinion impact in public health emergencies and conducted a configurational analysis, to reveal the generation mechanism of online public opinion impact. Up to now most studies have focused on the impact factors of online public opinion on public emergencies while there are few studies on the influence of online public opinion in public health emergencies (47). Public health emergencies as a special area of emergencies, because they have a real impact on the safety of public life and health, are easy to attract public attention and trigger high impact online discussion. Therefore, our targeted analysis of such events has important theoretical implications for preventing and resolving major public opinion risks and reducing their impact.

Second, we contribute to information ecology theory. The generation of online public opinion impact is systematic and complex, and it is necessary to further analyze the synergistic impact of various factors such as information person, information, information technology, and information environment (48). On the basis of the four dimensions of the information ecology theory, we refine it and extract seven preconditions conditions affecting online public opinion in public health emergencies: internet user’s attention, opinion leader dissemination, government intervention, hazard level, network platform participation, government public opinion evacuation environment, and social opinion environment, revealing the interaction mechanism among them.

Third, we apply QCA method to online public opinion research, which broadens the choice of online public opinion research methods. The generation of public opinion impact conforms to the characteristics of multi-causal induction. This study not only reveals the multifactorial configuration of high-impact online public opinion, but also reveals the configuration leading to not high-impact online public opinion.

### 5.3 Practical implications

Public health emergencies are closely related to the interests of the public and can create a public opinion field where the discourse related to the event is gathered, released, contested and divided. The factors triggering the impact of online public opinion in public health emergencies are multiple and varied, and the paths are complicated, so preventing or reducing the public opinion risks implied by public opinion becomes a complex systematic project. Based on the generation mechanism and evolution characteristics of the impact of online public opinion in public health emergencies, the following risk avoidance measures are proposed: first, define the discourse space of online opinion leaders and maintaining rational communication order; second, improve the management system of network platforms and build new central media platforms; third, establish a collaborative information sharing mechanism and build a strong interactive public opinion ecology; fourth, build an institution for public opinion risk research and evaluation, improve the ability of government intervention and evacuation. This study analyzed the generation of online public opinion impact of public health emergencies, in order to provide new ideas and management pathways for future public health emergencies.

### 5.4 Limitations and future research

Despite our best efforts, our research still has limitations. First, we selected these cases for public health emergencies that all occurred in China. Affected by the time span of typical cases of public health emergencies and the development cycle of the Internet platform, many details of typical cases of different events, such as the number of materials, information quality, and development map, are inevitably lost and missed. Moreover, 40 typical cases may not be enough to cover all the factors of public health emergencies, which will lead to certain errors in the results. Additionally, if cases from other countries can be included in the study, we can further enrich our research results and enhance its universality. Second, although the cases selected were all public health emergencies, these events have different types and ripple effects. In the future, our study can be further refined by analyzing and comparing the differences in the influencing factors among them according to the types of events, which can be more focused. Third, this study only focused on the role of four dimensions of information person, information, information technology and information environment on the impact of online public opinion, a more comprehensive research model can be constructed based on this study by further subdividing the four dimensions in the future.

## Data Availability

The datasets presented in this study can be found in online repositories. The names of the repository/repositories and accession number(s) can be found in the article/supplementary material.

## References

[1] Cleaton JM Viboud C Simonsen L Hurtado AM Chowell G. Characterizing Ebola transmission patterns based on internet news reports. Clin Infect Dis. (2016) 62:24–31. doi: 10.1093/cid/civ748, PMID: 26338786 PMC4678106

[2] Habermas J. Communication and the Evolution of Society. Cambridge: Polity Press (1991).

[3] Huang Y Yu Z Guo J Yu Z Xian Y. Legal public opinion news abstractive summarization by incorporating topic information. Int J Mach Learn Cybern. (2020) 11:2039–50. doi: 10.1007/s13042-020-01093-8

[4] Ayers JW Althouse BM Dredze M Leas EC Noar SM. News and internet searches about human immunodeficiency virus after Charlie Sheen’s disclosure. JAMA Intern Med. (2016) 176:552–4. doi: 10.1001/jamainternmed.2016.0003, PMID: 26902971

[5] Panagiotopoulos P Barnett J Bigdeli AZ Sams S. Social media in emergency management: twitter as a tool for communicating risks to the public. Technol Forecast Soc. (2016) 111:86–96. doi: 10.1016/j.techfore.2016.06.010

[6] Lash S. Reflexive modernization: the aesthetic dimension. Theor Cult Soc. (1993) 10:1–23. doi: 10.1177/026327693010001001

[7] Cao Y Shan J Gong Z Kuang J Gao Y. Status and challenges of public health emergency management in China related to COVID-19. Front Public Health. (2020) 8:250. doi: 10.3389/fpubh.2020.00250, PMID: 32574311 PMC7273973

[8] Posid JM Bruce SM Guarnizo JT O’Connor RC Papagiotas SS Taylor ML. Public health emergencies and responses: what are they, how long do they last, and how many staff does your agency need? Biosecur Bioterror. (2013) 11:271–9. doi: 10.1089/bsp.2013.0044 24219494

[9] Fleming N. Fighting coronavirus misinformation. Nature. (2020) 583:155–6. doi: 10.1038/d41586-020-01834-3, PMID: 32601491

[10] Cochran JK Chamlin MB. Can information change public opinion? Another test of the Marshall hypotheses. J Crim Just. (2005) 33:573–84. doi: 10.1016/j.jcrimjus.2005.08.006

[11] Noelle-Neumann E. The spiral of silence a theory of public opinion. J Commun. (1974) 24:43–51. doi: 10.1111/j.1460-2466.1974.tb00367.x

[12] Zhang X Zhou Y Zhou F Pratap S. Internet public opinion dissemination mechanism of COVID-19: evidence from the Shuanghuanglian event. Data Technol Appl. (2022) 56:283–302. doi: 10.1108/DTA-11-2020-0275

[13] Gao G Wang T Zheng X Chen Y Xu X. A systems dynamics simulation study of online public opinion evolution mechanism. J Glob Inf Manag. (2019) 27:189–207. doi: 10.4018/JGIM.2019100110

[14] Xie T Wei Y Chen W Huang H. Parallel evolution and response decision method for public sentiment based on system dynamics. Eur J Oper Res. (2020) 287:1131–48. doi: 10.1016/j.ejor.2020.05.025, PMID: 32834432 PMC7244451

[15] Qu Y Tian H Chen H. Research on the emotional evolution mechanism of network public opinion based on an information ecosystem. Discret Dyn Nat Soc. (2022) 2022:4875099. doi: 10.1155/2022/4875099

[16] Davenport TH Prusak L. Information Ecology: Mastering the Information and Knowledge Environment. New York: Oxford University Press (1997).

[17] Nardi BA O'Day V. Information Ecologies: Using Technology With Heart. Massachusetts: MIT Press (1999).

[18] Finin T Joshi A Kolari P Java A Kale A Karandikar A. The information ecology of social media and online communities. AI Mag. (2008) 29:77–92. doi: 10.1609/aimag.v29i3.2158

[19] Kish K. Paying attention: big data and social advertising as barriers to ecological change. Sustainability. (2020) 12:1–27. doi: 10.3390/su12241058935136666

[20] Gernat T Rao VD Middendorf M Dankowicz H Goldenfeld N Robinson GE. Automated monitoring of behavior reveals bursty interaction patterns and rapid spreading dynamics in honeybee social networks. Proc Natl Acad Sci USA. (2018) 115:1433–8. doi: 10.1073/pnas.1713568115, PMID: 29378954 PMC5816157

[21] Kim D Eom K Chung CJ. Measuring online public opinion for decision making: application of deep learning on political context. Sustainability. (2022) 14:1–16. doi: 10.3390/su14074113

[22] Enke N Borchers NS. Social media influencers in strategic communication: a conceptual framework for strategic social media influencer communication. Int J Strateg Commun. (2019) 13:261–77. doi: 10.1080/1553118X.2019.1620234

[23] Chen T Wang Y Yang J Cong G. Modeling public opinion reversal process with the considerations of external intervention information and individual internal characteristics. Healthcare. (2020) 8:160. doi: 10.3390/healthcare8020160, PMID: 32517050 PMC7349120

[24] Boehmer J Tandoc EC. Why we retweet: factors influencing intentions to share sport news on twitter. Int J Sport Commun. (2015) 8:212–32. doi: 10.1123/IJSC.2015-0011

[25] Liu J Liu L Tu Y Li S Li Z. Multi-stage internet public opinion risk grading analysis of public health emergencies: an empirical study on microblog in COVID-19. Inf Process Manag. (2022) 59:102796. doi: 10.1016/j.ipm.2021.102796, PMID: 34744256 PMC8556697

[26] Xing Y Li Y Wang FK. How privacy concerns and cultural differences affect public opinion during the COVID-19 pandemic: a case study. Aslib J Inf Manag. (2021) 73:517–42. doi: 10.1108/AJIM-07-2020-0216

[27] Zhang M Su H Wen J. Analysis and mining of internet public opinion based on LDA subject classification. J Web Eng. (2021) 20:2457–72. doi: 10.13052/jwe1540-9589.20811

[28] Gerbaudo P Trere E. In search of the “we” of social media activism: introduction to the special issue on social media and protest identities. Inf Commun Soc. (2015) 18:865–71. doi: 10.1080/1369118X.2015.1043319

[29] Cervi L García F Lladó CM. Populism, twitter, and covid-19: narrative, fantasies, and desires. Sociol Sci. (2021) 10:294. doi: 10.3390/socsci10080294

[30] Jarynowski A Belik V Wójta-Kempa M. Trends in interest of COVID-19 on polish internet. Przegl Epidemiol. (2020) 74:258–75. doi: 10.32394/pe.74.20, PMID: 33112108

[31] Beckers K. Power of the people or the expert? The influence of vox pop and expert statements on news-item evaluation, perceived public opinion, and personal opinion. Commun Ger. (2022) 47:114–35. doi: 10.1515/commun-2019-0186

[32] Leong AD Ho SS. Perceiving online public opinion: the impact of Facebook opinion cues, opinion climate congruency, and source credibility on speaking out. New Media Soc. (2021) 23:2495–515. doi: 10.1177/1461444820931054

[33] Yin F Shao X Wu J. Nearcasting forwarding behaviors and information propagation in Chinese Sina-microblog. Math Biosci Eng. (2019) 16:5380–94. doi: 10.3934/mbe.2019268 31499717

[34] Wang G Zhang W Zeng R. WeChat use intensity and social support: the moderating effect of motivators for WeChat use. Comput Hum Behav. (2019) 91:244–51. doi: 10.1016/j.chb.2018.10.010

[35] Wen Z Geng X Ye Y. Does the use of WeChat Lead to subjective well-being? The effect of use intensity and motivations. Cyberpsychol Behav Soc Netw. (2016) 19:587–92. doi: 10.1089/cyber.2016.0154, PMID: 27732075

[36] Liu Y Wu Y Li C Song C Hsu W. Does displaying one’s IP location influence users’ privacy behavior on social media? Evid Chin Weibo Telecommun Policy. (2024) 48:102759. doi: 10.1016/j.telpol.2024.102759

[37] Gao S Zhang Y Liu W. How does risk-information communication affect the rebound of online public opinion of public emergencies in China? Int J Environ Res Public Health. (2021) 18:7760. doi: 10.3390/ijerph18157760 34360053 PMC8345355

[38] Ragin CC Fiss PC. Net Effects Analysis versus Configurational Analysis: An Empirical Demonstration. Redesigning Social Inquiry: Fuzzy Sets and Beyond. Chicago: University of Chicago Press (2008).

[39] Fiss PC. Building better causal theories: a fuzzy set approach to typologies in organization research. Acad Manag J. (2011) 54:393–420. doi: 10.5465/AMJ.2011.60263120

[40] Schneider CQ Wagemann C. Standards of good practice in qualitative comparative analysis (QCA) and fuzzy-sets. Comp Sociol. (2010) 9:397–418. doi: 10.1163/156913210X12493538729793

[41] Du Y Kim PH. One size does not fit all: strategy configurations, complex environments, and new venture performance in emerging economies. J Bus Res. (2021) 124:272–85. doi: 10.1016/j.jbusres.2020.11.059

[42] Du Y Kim PH Aldrich HE. Hybrid strategies, dysfunctional competition, and new venture performance in transition economies. Manag Organ Rev. (2016) 12:469–501. doi: 10.1017/mor.2016.30

[43] Hudson J Kühner S. Qualitative comparative analysis and applied public policy analysis: new applications of innovative methods. Polic Soc. (2013) 32:279–87. doi: 10.1016/j.polsoc.2013.10.001

[44] De Crescenzo V Ribeiro-Soriano DE Covin JG. Exploring the viability of equity crowdfunding as a fundraising instrument: a configurational analysis of contingency factors that lead to crowdfunding success and failure. J Bus Res. (2020) 115:348–56. doi: 10.1016/j.jbusres.2019.09.051

[45] Rihoux B Ragin CC. Configurational Comparative Methods: Qualitative Comparative Analysis (QCA) and Related Techniques. London: SAGE Publications (2008).

[46] Greckhamer T Furnari S Fiss PC Aguilera RV. Studying configurations with qualitative comparative analysis: best practices in strategy and organization research. Strateg Organ. (2018) 16:482–95. doi: 10.1177/1476127018786487

[47] Hui F. Research on the construction of emergency online public opinion emotional dictionary based on emotional feature extraction algorithm. Front Psychol. (2022) 13:857769. doi: 10.3389/fpsyg.2022.857769, PMID: 35529545 PMC9072777

[48] Depoux A Martin S Karafillakis E Preet R Larson HJ. The pandemic of social media panic travels faster than the COVID-19 outbreak. J Travel Med. (2020) 27:1195–982. doi: 10.1093/jtm/taaa031 PMC7107516 32125413

